# Hydrops regression after vestibular denervation - longitudinal magnetic resonance study in patients with severe Meniere’s disease treated with vestibular neurectomy

**DOI:** 10.1007/s13760-024-02605-x

**Published:** 2024-07-30

**Authors:** Agnieszka Jasińska-Nowacka, Magdalena Lachowska, Emilia Wnuk, Kazimierz Niemczyk

**Affiliations:** 1 Department of Otorhinolaryngology Head and Neck Surgery, Medical University of Warsaw, ul. Banacha 1a, Warszawa (Warsaw), 02-097 Poland; 2 Department of Clinical Radiology, Medical University of Warsaw, Warsaw, Poland

**Keywords:** Endolymphatic hydrops, Vertigo, Hearing loss, Vestibular neurectomy

## Abstract

**Objectives:**

The aim was to evaluate endolymphatic hydrops in patients with severe Ménière’s disease (MD) before and after vestibular neurectomy to verify if vestibular denervation results in hydrops regression.

**Methods:**

Magnetic resonance imaging was performed after intravenous gadolinium injection in twenty patients with unilateral definite MD before and after the vestibular neurectomy. Clinical symptoms and audiovestibular tests were evaluated. Follow-up intervals ranged from 18 to 35 months after the surgery.

**Results:**

Endolymphatic hydrops were visualized in all patients in the preoperative scans. After the vestibular neurectomy, all patients presented a complete resolution of vertigo episodes. Regression of the endolymphatic hydrops was observed in 35% and 15% of cases analyzing cochlea and vestibule, respectively. In 71.43% of patients with utricular herniation into the lateral semicircular canal, withdrawal of the hernia was visualized. Asymmetrical contrast enhancement in the cochlea regressed in 17.64% of cases. Analyzing all the parameters collectively, in 60% of patients, partial regression of at least one of the radiological signs was confirmed in the follow-up examination. No progression of the endolymphatic hydrops was visualized after the surgery in either the cochlea or the vestibule.

**Conclusions:**

Vestibular neurectomy is an effective treatment, eliminating vertigo attacks and improving the quality of life in patients with MD. Magnetic resonance imaging of the inner ear allows visualization of changes in endolymphatic hydrops degree after treatment. Regression of the endolymphatic hydrops after vestibular neurectomy suggests that vestibular denervation may effectively halt the progression of the endolymphatic space dilatation and result in hydrops regression.

## Introduction

Ménière’s disease (MD) is a chronic inner ear condition characterized by vertigo attacks accompanied by tinnitus, aural fullness, and sensorineural hearing loss. Although Prosper Ménière described the typical clinical manifestation in 1861 [1], it took almost eighty years to visualize the underlying pathology causing MD. In 1938, Hallpike & Cairns and independently Yamakawa [2, 3] studied temporal bones of patients with MD symptoms and described dilatation of the endolymphatic space. Since then, it has been possible to confirm endolymphatic hydrops (EH), but only in the postmortem histopathological examination. Nowadays, visualization of the inner ear in magnetic resonance imaging (MRI) allows us to confirm EH in vivo. It was reported by Nakashima et al. in 2007 [4], and since then, subsequent studies have explored various protocols for EH visualization [4–8]. Until recently, evaluating the effectiveness of MD treatment was limited to the patient’s symptoms and audiovestibular test results because in vivo assessment of the inner ear structures was impossible. Repeated MRI examinations allow for assessing changes in EH degree after treatment, making it a valuable tool for follow-up evaluation in MD patients. Several studies concerning changes in EH after conservative and surgical treatment have been published in the last few years [7, 9–16]. However, to our best knowledge, no previous research has been conducted on long-term MRI follow-up after selective vestibular nerve section.

The present study analyzed MRI scans in patients with unilateral uncontrollable MD before and after the vestibular neurectomy. The objective was to visualize in vivo morphological changes occurring in the inner ear after vestibular denervation. In particular, we aimed to assess changes in EH degree after the surgery and to explore correlations between the MRI results and the clinical course of MD.

## Materials and methods

### Ethical consideration

The study was conducted with the approval of the Institutional Ethics Committee (KB/110/2019) and was performed following the Declaration of Helsinki. All patients provided written informed consent before participating in the study.

### Patients description

Twenty patients diagnosed with unilateral defined MD according to the 1995 and 2020 guidelines of the Committee of Hearing and Equilibrium American Academy of Otolaryngology, Head and Neck Surgery (AAO-HNS) [17] and Barany Society criteria [18] were enrolled in this study. The patients suffered from intractable MD, defined as recurrent vertigo attacks, despite a minimum of six months of conservative treatment, including appropriate life guidance (i.e., low-salt diet, stress reduction, adequate water intake), oral therapy with betahistine (48 mg a day), diuretic drugs and intratympanic steroid injections (2 courses with four injections each). Exclusion criteria were as follows: bilateral clinical symptoms of MD, age > 65 years, past medical history of chronic otitis media or otosclerosis, prior history of ear surgery or ototoxic therapy including intratympanic gentamicin, MRI-related contraindications, and chronic diseases contraindicating general anesthesia. The decision of vestibular neurectomy was made individually for each patient due to the uncontrollable clinical course, persistent symptoms, functional impairment defined as a minimum level of 4 on the AAO–HNS scale, and/or Tumarkin’s drop attacks, causing significant limitations in daily life activities.

### Clinical symptoms and audiological test evaluation protocol

The main clinical symptoms were evaluated during the diagnostic hospitalization. A retrospective questionnaire was used to enable patients to self-assess the intensity of their symptoms during the past six months. An average frequency of vertigo attacks per month was calculated. The average intensity of tinnitus, aural fullness, and balance problems was assessed using a scale from 0 to 6 proposed by Arenberg and Stahle [19]. The functional level was self-estimated by the patients using a scale ranging from 1 to 6 established by AAO-HNS, where level 1 represents no impact of the disease on daily activities, and the severity of symptoms increases with the scale up to level 6, which represents an inability to work for more than one year [17]. The same protocol was conducted during the follow-up visit 18 to 35 months after the surgery.

Standard pure-tone and speech audiometry were performed during the preoperative diagnostic hospitalization and repeated during the follow-up visit. The pure tone average (PTA) hearing levels were calculated as the mean values among air-conduction hearing threshold levels at 500, 1000, 2000, and 4000 Hz. The hearing threshold level at a low frequency of 250 Hz was also evaluated. Changes between pre- and postoperative hearing levels of more than 10 dB were considered significant. In speech audiometry, analysis word recognition score (WRS) was evaluated.

### Surgical treatment

In all patients, a very experienced otosurgeon (one of the authors of this manuscript) performed the vestibular nerve section. Surgery was performed through the middle fossa approach with the vestibular ganglion’s removal and the cochlear nerve’s preservation.

### MRI assessment protocol

The MRI scans were performed in all twenty patients before and after vestibular neurectomy using the same protocol described by Wnuk et al. [20]. A 3 Tesla MR scanner (Signa Architect, GE Healthcare, Milwaukee, WI, USA) with a 16-channel phased array flex coil (GEM Flex Large coil, Neocoil, Pewaukee, WI, USA) was used. The sequence was characterized by the following parameters: 3D-FLAIR with a fat-suppression, acquired on an axial plane, covering the posterior cranial fossa; time of repetition 7602 ms, time of echo 170 ms, time of inversion 1897 ms, NEX 2.0, the field of view 18, slice thickness 0.8 mm, variable flip angle. A double dose (0.2 mL/kg) of intravenously injected gadobutrol (Gadovist; Bayer Schering Pharma AG, Berlin, Germany; 1.0 mmol/mL) was used to achieve optimal perilymphatic enhancement, and the dedicated sequence was performed 4 h after administration of the contrast agent, as explained by Wnuk et al. [20, 21]. Gadolinium accumulated in the perilymph while the endolymphatic space was visualized as a partial loss of contrast enhancement. The cochlear EH was evaluated using the Barath scale [5], and the vestibular EH was assessed using the Bernaerts grading scale [6], which modified the Barath classification.

Moreover, utricular herniation of the endolymphatic space into the lateral semicircular canal was assessed regarding the three-dimensional fast inflow steady-state acquisition (3D-FIESTA) sequence visualizing the inner ear fluid space. Additionally, as proposed by Bernaerts et al. [6], the contrast enhancement of the cochlea in the affected ear was assessed and compared to the asymptomatic opposite side. The same MRI assessment protocol was used and verified by Jasińska et al. [22], with 94.7% sensitivity in confirming MD.

### Statistical analysis

Statistical analysis was conducted in the STATISTICA program (TIBCO Software Inc. 2017, version 13.3). The data were tested for normality, parametric, and non-parametric criteria. Detailed statistical analysis was performed using the Mann-Whitney U and Wilcoxon signed-rank tests. The level of statistical significance was set at *p* = 0.05.

## Results

### Patients’ characteristics

Twenty patients (13 females and 7 males) with unilateral definite MD (13 in the left ear, 7 in the right ear) were enrolled in this study. The age of the population ranged from 34 to 65 years (mean 50.60 years). Disease duration ranged from 1 to 18 years (mean 6.93), analyzing the period from the first MD symptom. In contrast, considering the time from the first characteristic vertigo attack, the average duration of this period was 5.60 years, as in 65% of patients MD started with isolated aural symptoms preceding vertigo attack.

The clinical profiles of all patients, including pre- and postoperative clinical data, audiological results, and EH degree, are presented in Table 1.

**Table 1:** Clinical, audiological, and MRI characteristics of the analyzed patients with unilateral definite Ménière’s disease before and after vestibular neurectomy from the middle fossa approach. MD - Ménière’s disease; AAO – HNS- American Academy of Otolaryngology-Head and Neck surgery; MRI - magnetic resonance imaging; PTA - pure tone average; *- unreliable assessment due to the increased perilymphatic enhancement

Patient	Age (years)	Gender (M- male, F- female)	Affected side (R-right, L-left)	MD duration (years)	Functional level 1–6 AAO-HNS scale (pre/postoperative)	PTA hearing level (dBHL) (pre/postoperative)	Follow-up interval (months)	MRI endolymphatic hydrops evaluation (pre/postoperative)				
								cochlea (Barath scale)	vestibule (Bernaerts scale)	utricle herniation into lateral SCC	Increased contrast enhancement	
											cochlea	vestibule
#1	55	M	L	3	6 / 1	35.00 / 61.25	28	1 / 1	2 / 2	0 / 0	1 / 0	0 / 0
#2	65	F	L	2	5 / 2	68.75 / 88.75	23	2 / 2	2 / *	0 / *	1 / 1	0 / 1
#3	65	F	L	10	5 / 1	71.25 / 68.75	26	2 / 1	2 / 2	0 / 0	1 / 1	0 / 0
#4	49	F	L	13	3 / 1	67.50 / 47.50	35	2 / 0	1 / *	0 / *	0 / 1	0 / 1
#5	62	F	L	7	5 / 2	57.50 / 60.00	24	2 / 2	3 / 3	1 / 1	1 / 1	0 / 0
#6	51	F	R	11	4 / 2	43.75 / 42.50	23	1 / 0	2 / *	0 / *	1 / 1	0 / 1
#7	35	F	R	18	5 / 2	32.50 / 41.25	23	1 / 1	3 / 2	1 / 0	1 / 0	0 / 0
#8	56	F	R	1	5 / 1	70.00 / 78.75	22	2 / 2	3 / 1	1 / 0	1 / 1	0 / 0
#9	61	F	L	11	5 / 1	32.50 / 73.75	25	1 / 0	3 / 2	1 / 0	1 / 1	0 / 0
#10	39	F	R	8	5 / 2	63.75 / 108.75	18	1 / 1	2 / 2	0 / 0	1 / 1	0 / 0
#11	61	F	L	12	3 / 2	78.75 / 100.00	28	1 / 0	3 / *	1 / *	1 / 1	0 / 1
#12	65	F	L	9	3 / 3	66.25 / 70.00	27	1 / 1	3 / 3	1 / 0	1 / 1	0 / 0
#13	60	F	L	2	5 / 2	26.25 / 65.00	27	1 / 1	2 / *	0 / 0	0 / 1	0 / 1
#14	59	M	R	3	5 / 1	40.00 / 52.50	26	1 / 1	2 / *	0 / *	1 / 0	0 / 1
#15	45	M	R	5	4 / 1	72.50 / 97.50	28	2 / 0	3 / 3	1 / 0	1 / 1	0 / 0
#16	39	M	L	9	5 / 1	68.75 / 60.00	31	2 / 2	3 / *	0 / 0	1 / 1	0 / 1
#17	38	F	L	3	5 / 4	48.75 / 57.50	22	2 / 2	2 / 2	0 / 0	0 / 0	0 / 0
#18	36	M	L	3	4 / 1	48.75 / 120.00	21	1 / 0	2 / *	0 / *	1 / 1	0 / 1
#19	34	M	L	1,5	4 / 1	50.00 / 56.25	21	2 / 2	2 / *	0 / *	1 / 1	0 / 1
#20	37	M	R	7	4 / 1	17.50 / 50.00	24	1 / 1	2 / 2	0 / 0	1 / 1	0 / 0

### Preoperative clinical data

The mean frequency of vertigo episodes within six months before surgery was 2.93 per month, and 20% of patients suffered from Tumarkin’s drop attacks. The mean severity of tinnitus, aural fullness, and balance problems were 4.36, 3.02, and 3.26, respectively, on the 0–6 Arenberg scale. The average functional level was 4.50 on the 1–6 AAO-HNS scale, and all the patients self-assessed their level of disability associated with MD, ranging from 4 to 6. The patients denied aural symptoms in the opposite ear associated with vertigo episodes.

### Preoperative audiological results

In pure-tone audiometry, pantonal sensorineural hearing loss, defined as a PTA level above 20 dB HL with hearing thresholds of at least 25 dB HL for frequencies 500, 1000, 2000, and 4000 Hz, and no air-bone gap, was found in 90% of patients. 5% of patients (one patient, #13) had low-frequency hearing loss affecting frequencies of 250 Hz, 500 Hz, and 1000 Hz with a PTA value of 26.25 dB HL. 5% of patients (one patient, #20) presented a normal PTA value of 17.50 dB HL, although the hearing threshold levels in low and mid frequencies were 40 dB HL for 250 Hz and 500 Hz.

Considering all patients as a population, the mean PTA level was 53 dB HL (± 17.95 dB) in the affected ears and 16.54 dB HL (± 10.28 dB) in the contralateral unaffected ears. The average pre- and postoperative hearing threshold levels are presented in Table 1.

In speech audiometry, a 50% word recognition level was achieved by 65%, whereas 100% word discrimination was achieved by 30% of patients. The mean WRS was 67.5%. The average WRS in the contralateral ear was 95%.

### Preoperative MRI results

In all patients, the preoperative MRI examination revealed enlarged endolymphatic space in the cochlea and the vestibule (Table 1). In the cochlea, EH grade 1 was visualized in 55% of patients, while EH grade 2 was present in 45%. Analyzing vestibular endolymphatic space with the Barath scale, EH grade 1 was present in 55% of patients, while EH grade 2 was observed in 40%. In 5% of patients (one patient, #4), vestibular endolymphatic space did not meet the Barath criteria for hydrops. However, according to the modified grading scale proposed by Bernaerts et al. [6], it was classified as extra-low grade 1 of the vestibular EH as the saccule was larger than the utricle.

**Fig. 1:**
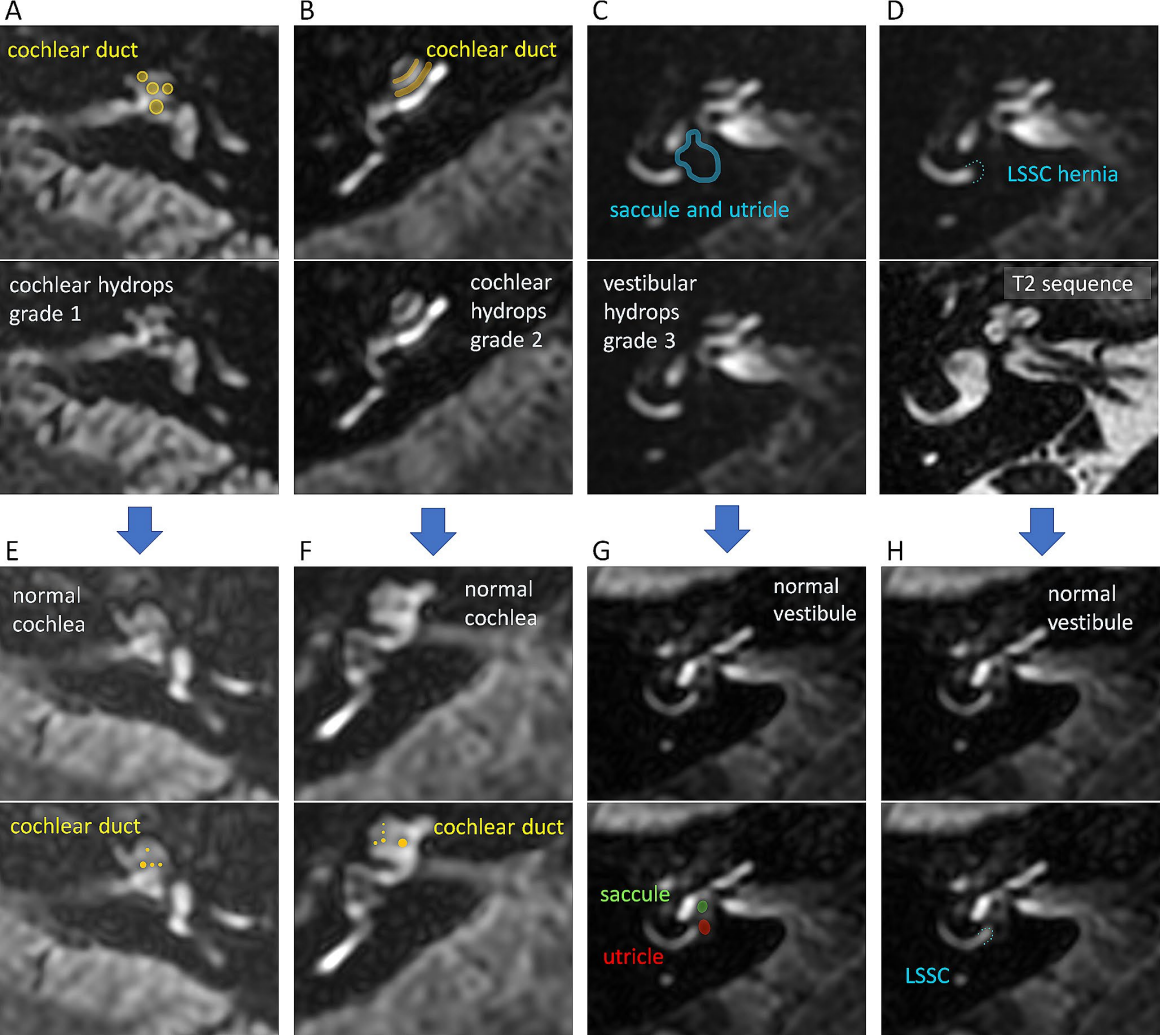
Magnetic resonance imaging (MRI) of patients with clinically diagnosed definite Ménière’s disease before and after vestibular neurectomy (axial plane, delayed Gd-enhanced images). Panels A-D show the preoperative MRI scans: (**A**) a dilated cochlear duct with partially obliterated scala vestibuli - grade 1 of the cochlear hydrops according to the Barath scale; (**B**) A dilated cochlear duct with fully obliterating scala vestibuli - grade 2 of the cochlear hydrops according to the Barath scale; (**C**) A dilated saccule and utricle occupying all the vestibule – vestibular hydrops grade 3 according to Bernaerts scale; and (**D**) Utricular herniation into the lateral semicircular canal (LSCC). 3D Heavily T2-weighted sequence visualizing preserved fluid signal. The upper images on panels A-D show the same images as the lower ones in respective panels but in the form of companion scans with some line art applied to define specific anatomical structures for easier identification of observed pathologies. Panels E-H show the postoperative MRI scans: (**E**) and (**F**) Complete regression of the cochlear endolymphatic hydrops, the narrow cochlear duct barely visible between the scala tympani and scala vestibuli; (**G**) Complete regression of the vestibular endolymphatic hydrops, saccule and utricle visualized as two separated, non-enhanced endolymphatic structures; (**H**) Withdrawal of the utrical hernia into the lateral semicircular canal (LSCC), the posterior arm of the LSCC confluent with the vestibular perilymphatic space. The bottom images on panels E-H show the same images as the upper ones in respective panels but in the form of companion scans with some line art applied to define specific anatomical structures for easier identification of observed pathologies

In 35% of patients, the endolymphatic space of the vestibule herniated into the lateral semicircular canal (Fig. 1D). In these cases, the endolymphatic space fully occupied the vestibule (EH grade 3 according to the Bernaerts scale).

**Fig. 2:**
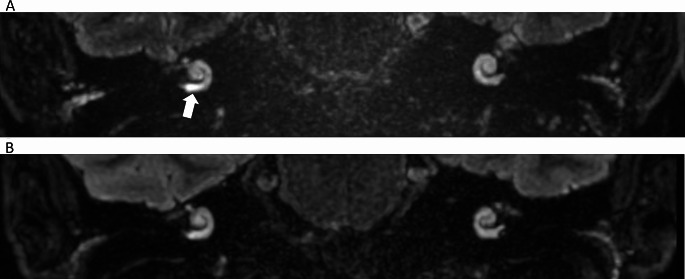
Magnetic resonance imaging (MRI) of a patient with a clinical diagnosis of definite Ménière’s disease in the right ear (case #14) before and after vestibular neurectomy (coronal plane, delayed Gd-enhanced images). Panel (**A**) A moderate asymmetrical perilymphatic enhancement in the basal turn of the cochlea (pointed by the dotted arrow) visualized preoperatively, indicating a temporary increase of the blood-perilymph barrier permeability; Panel (**B**) Regression of the asymmetrical perilymphatic enhancement visualized in the scan 2.16 years after the surgery

According to the Bernaerts grading system, asymmetrical contrast enhancement of the inner ear in the affected site was described in 85% of patients (Fig. 2A**)**.

No cochlear endolymphatic hydrops were found among analyzed patients in the contralateral unaffected ears. After evaluating MR images with the Barath scale, no endolymphatic hydrops were also found in the vestibule in the contralateral ears. However, in three patients (#5, #10, #19), the contralateral ear’s saccule was larger than the utricle without the confluence between these structures. Thus, upon evaluation using the Bernaerts scale, these results were described as extra-low grade 1 of the vestibular EH despite not meeting the Barath criteria. None of these patients reported aural symptoms in the contralateral ear.

### Postoperative clinical data

The patients were discharged home on the seventh day after the neurectomy as the acute vertigo after the surgical treatment subsided. During the hospitalization, all patients received steroids after the surgery to avoid facial nerve palsy and edema. Besides vertigo, no acute postoperative complications occurred among the analyzed patients. No facial nerve dysfunction was present when patients were discharged to home.

The follow-up visit took place on average 25.5 months after the vestibular neurectomy (18 to 35 months). All patients reported total resolution of vertigo episodes with no attack during at least the last six months before the follow-up visit (class A vertigo control according to the AAO-HNS criteria). None of the patients suffered from Tumarkin drop attacks after the surgery. Six months before the follow-up visit after the neurectomy, the mean severity of aural symptoms decreased compared to the six months before the surgery. Moreover, the average tinnitus, aural fullness, and balance problems have been reduced to 3.48, 1.12, and 1.55, respectively, on the 0–6 Arenberg scale. In 95% of patients, functional levels improved after the surgery, while 5% of patients (one patient, #12) reported a stable functional status of grade 3 on the 1–6 AAO-HNS scale. During the follow-up visit, the average functional level was 2.00, with 95% of the patients self-assessing their postoperative disability level as grade 1 to 3 and 5% of patients (one patient, #17) as grade 4 (Table 1). At the follow-up visit, mild dysfunction of the facial nerve (level II on the House-Brackmann scale) was present in two patients (#2 and #5) [23].

### Postoperative audiological results

After the surgery, the average PTA level in the affected ears was 70 dB HL (± 22.41 dB), with an average deterioration of 17 dB. Moreover, each analyzed frequency’s mean hearing threshold level worsened. Among twenty patients, the PTA level remained stable in 50% of cases, and significant hearing deterioration occurred in 45%. In contrast, in 5% (one patient, #4), hearing improvement of more than 10 dB was observed (Table 1). The average PTA level in the contralateral unaffected ears was 17.38 dB HL (± 11.60 dB), with no significant change in the PTA level compared to the preoperative examination.

At the follow-up visit, 50% of patients achieved a word recognition level of 50%, while 100% achieved a word discrimination level of 20%. The mean WRS was 54.74%. The average WRS in the contralateral ear was 95.53%.

### Postoperative MRI results

Detailed analysis of the endolymphatic space changes in the postoperative MRI scans of each patient is presented in Table 1. No progression of the EH was visualized after the surgery in either the cochlea or the vestibule.

In the cochlea, regression of the EH was confirmed in 35% of cases - partial reduction of the EH was observed in 5%, while complete withdrawal of the EH was visualized in 30% of patients. In 10% of patients (#4, #15), severe cochlear EH grade 2 resolved completely in the postoperative examination (Table 1; Fig. 1B and F). Postoperative reduction of the cochlear EH degree was statistically significant (*p* = 0.02).

**Fig. 3:**
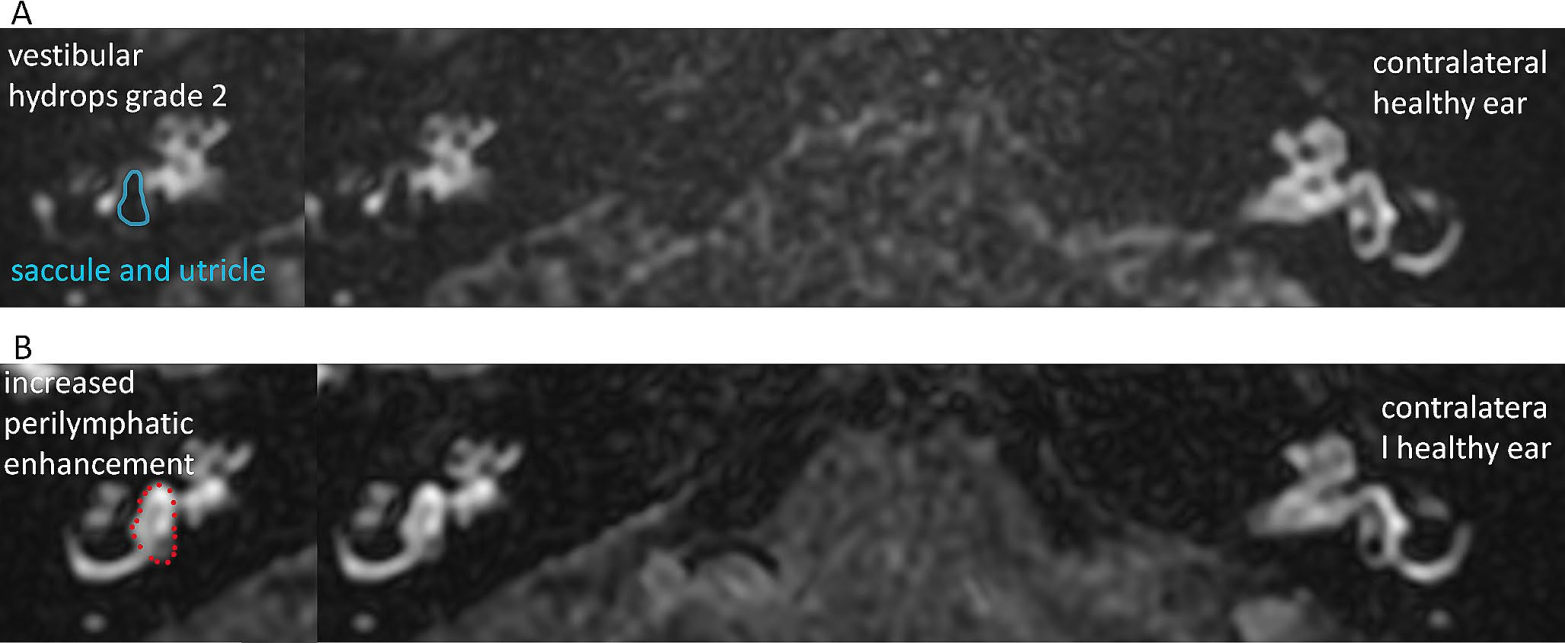
Magnetic resonance imaging (MRI) of a patient with a clinical diagnosis of definite Ménière’s disease in the right ear (case #14) before and 2.16 years after vestibular neurectomy (axial plane, delayed Gd-enhanced images). Panel (**A**) Preoperative images showing dilated saccule confluent with utricle with circular enhancing perilymphatic space – vestibular endolymphatic hydrops grade 2 according to Bernaerts scale; Panel (**B**) A severe asymmetrical perilymphatic enhancement in the vestibule preventing a reliable vestibular endolymphatic space evaluation in the postoperative examination. On both panels, the small left side images show the same images of the right analyzed ears, respectively, in the form of companion scans with some line art applied to define specific anatomical structures for easier identification of observed pathologies

In the vestibule, partial regression of the EH severity was visualized in 15% of patients. In these three patients, severe vestibular EH grade 3 was confirmed preoperatively (Fig. 1C and G). However, no complete reduction of the vestibular EH was confirmed in any of the cases. In 40% of patients, no change was observed in the vestibular MRI. Analyzing postoperative MRI scans in the remaining 45% of patients, severe increased contrast enhancement in the vestibule was visualized (Fig. 3), looking like an edematous perilymphatic space. Since it was present at all levels of MRI scans, it covered the entire vestibule with barely visible non-enhanced endolymphatic structures. Moreover, this phenomenon made selecting the optimal vestibular assessment level difficult. Thus, increased perilymphatic enhancement in the vestibule prevented a reliable vestibular EH degree evaluation. Considering only 11 patients in whom postoperative MRI vestibule assessment was possible, the EH severity change was not statistically significant (*p* = 0.11).

Analyzing EH in the cochlea and the vestibule collectively, in 45% of patients, partial regression of the hydrops was observed after the surgery. Still, none of the patients’ EH resolved completely in the affected inner ear.

In 71.43% of patients with utricular herniation into the lateral semicircular canal (5 out of 7 patients with hernia), withdrawal of the hernia was visualized in the follow-up MRI (Fig. 1D and H).

Among the patients with asymmetrical contrast enhancement in the affected ear, the postoperative MRI revealed a reduction of this phenomenon in 17.64% of cases (3 out of 17) (Fig. 2**)**. However, in 2 patients (#4, #13), increased contrast enhancement in the cochlea occurred in the follow-up examination despite the absence of that pathology in the preoperative scans, and both of these patients presented increased perilymphatic enhancement in the vestibule after the surgery (Table 1).

By comprehensively analyzing all the parameters mentioned above, in 60% of patients, partial regression of at least one of the radiological MD signs was confirmed in the follow-up examination.

Moreover, radiological signs of MD proposed by Bernaerts were evaluated collectively on a scale from 0 to 6 as follows: cochlear EH degree (0–2 points), vestibular EH degree (0–3 points), asymmetrical contrast enhancement in the cochlea (0–1 point). Then, analyzing regression of the MD radiological signs proposed by Bernaerts, differences between pre- and postoperative results were statistically significant (*p* = 0.03).

Additionally, we proposed a scale from 0 to 7 considering utricular herniation into the lateral semicircular canal as an additional point in the vestibular EH degree. Then, regression of the radiological signs of MD between pre- and postoperative MRI examinations was statistically significant (*p* = 0.02).

In the contralateral ears, no change in the endolymphatic space was observed in the follow-up MRI examinations.

### Correlation between postoperative MRI results and clinical data

**Fig. 4:**
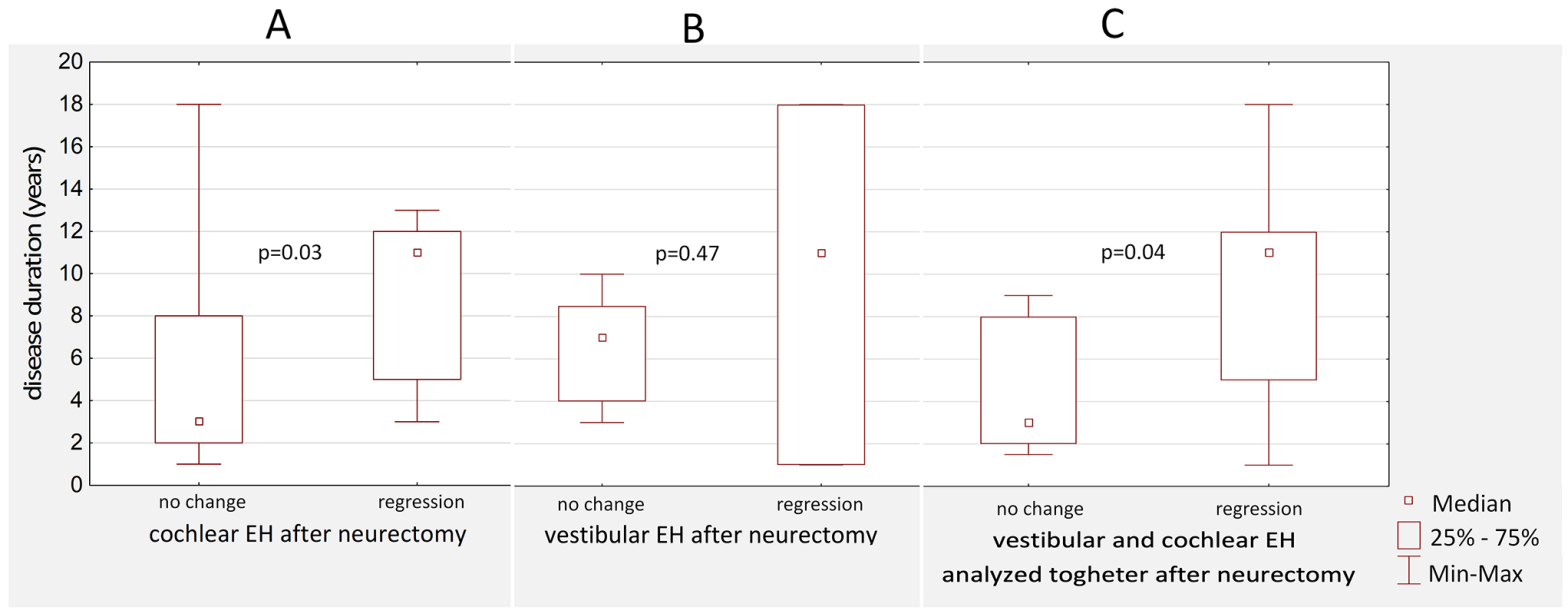
Differences in disease duration between patients with and without endolymphatic hydrops (EH) regression after vestibular neurectomy regarding cochlear endolymphatic hydrops analyzed alone (Panel A), vestibular endolymphatic hydrops analyzed alone (Panel B), and endolymphatic hydrops in the cochlea and the vestibule collectively (Panel C). *P* < 0.05 represents statistically significant differences

To analyze relationships between changes in cochlear EH severity and patients’ clinical characteristics (age, disease duration, follow-up interval, pure-tone audiometry results), the studied population was divided into two groups – patients with postoperative regression of cochlear EH and patients with no change in cochlear EH degree. Patients were divided into groups with and without vestibular EH regression to investigate changes in vestibular EH. Then, to analyze the change in vestibular and cochlear EH collectively, patients with regression of EH in at least one inner ear compartment and patients without change in EH were distinguished. Detailed results regarding the differences between these groups are presented in Fig. 4.

The patients with EH regression and patients with no change in EH degree (analyzing cochlear and vestibular EH separately and collectively) did not differ significantly (*p* > 0.05) in terms of age, time interval from the vestibular neurectomy to the follow-up MRI examination and preoperative vestibular and cochlear EH degree.

Analyzing changes in cochlear EH, the disease duration was significantly longer in patients with postoperative regression of EH (mean 9.29 years) compared to the group with no changes in EH degree (mean 5.65 years), with *p* = 0.03 (Fig. 4A). Moreover, the time from the first vertigo attack to vestibular neurectomy differed significantly (*p* = 0.01) between these two groups (mean 8.14 and 4.23 years, respectively). However, analyzed groups did not differ regarding pure-tone audiometry results, including pre- and postoperative PTA levels, hearing threshold levels for each analyzed frequency separately, and postoperative change in PTA level.

Regarding changes in vestibular EH, no statistically significant difference in the abovementioned clinical aspects was found between patients with postoperative regression of EH and the group with no changes in EH degree (Fig. 4B).

Analyzing changes in EH in the cochlea and the vestibule collectively, the disease duration (Fig. 4C) and time interval from the first vertigo attack were significantly longer in patients with postoperative regression of EH (*p* = 0.04 and *p* = 0.01, respectively). No statistically significant difference in other abovementioned clinical aspects was found between these groups.

Furthermore, the subpopulation with postoperative increased perilymphatic enhancement in the vestibule did not show significant clinical differences in the abovementioned variables compared to the others.

## Discussion

Despite numerous studies, the precise etiology of MD symptoms remains unclear, with genetic, autoimmune, and inflammatory factors being considered [24–29]. Consequently, the treatment of MD is mostly symptomatic and consists of pharmacological and surgical methods. According to the AAO-HNS guidelines, escalation of treatment interventions is recommended after analysis of the patient’s symptoms severity and audiological and vestibular test results [17, 30]. The non-invasive treatment provides a satisfactory level of vertigo control in 80–90% of cases, while the remaining 10–20% of patients may require surgical procedures [31–33].

Patients’ clinical improvement remains the most important factor in evaluating MD treatment, as the main goal of the therapy is to reduce the frequency and intensity of disabling vertigo attacks [17, 18, 30]. Vestibular neurectomy is indicated in cases with unilateral advanced MD with no clinical improvement after pharmacological and intratympanic treatment, as well as in patients with Tumarkin’s drop attacks in their history [17, 30, 34]. It is an effective method with complete vertigo episode resolution in over 90% of patients [35–37]. In the present study, all the patients reported complete resolution of vertigo attacks after the vestibular neurectomy, consistent with the literature [35, 37, 38]. Moreover, 95% of patients reported improved functional levels. As 95% of patients self-assessed their postoperative functional level as grade 1 to 3 in the 1–6 AAO-HNS scale, it may be presumed they could work or drive a car after the surgery, which confirms this method’s clinical effectiveness. However, the morphological and pathophysiological consequences of vestibular denervation underlying symptoms reduction or resolution remain unclear.

MRI of the inner ear has been used to diagnose MD for the last several years, and its sensitivity in EH visualization has been confirmed in numerous studies [5, 6, 21, 22]. Moreover, it may be a helpful tool in studying the natural course of MD due to the possibility of repeated examinations in the same patient [9, 11, 12]. Recently, studies regarding the dynamics of EH after various treatment methods were published [7, 9–16, 39]. It provides new opportunities to evaluate the effectiveness of conservative therapy and surgical interventions. Moreover, the possibility of in vivo inner ear visualization may provide information about EH dynamics and its correlations with the clinical outcome.

Jasińska-Nowacka et al. [39] described four patients who underwent a follow-up MRI eight months after the neurectomy, and no change in EH degree was visualized in these cases. To our best knowledge, our study is the first to investigate the change in EH after vestibular neurectomy in long-term observation, that is, over 1.5 years after.

In this study, with follow-up over 1.5 years after neurectomy, regression of the EH was observed in 35% and 15% of cases analyzing cochlear and vestibular EH, respectively. The section of the vestibular efferent fibers may result in partial regression of the EH after vestibular neurectomy, followed by the dark cells’ denervation, as was hypothesized before [39]. Then, due to the stria vascularis denervation, a decreased endolymph production in the cochlea would reduce the cochlear duct volume. Hypothetically, the withdrawal of the EH would occur successively in different parts of the inner ear as it occurs during the EH development – initially in the cochlea, then in the vestibule [40, 41]. That would explain why cochlear EH regressed in more patients than vestibular EH.

Interestingly, in our study, no change in cochlear EH was visualized in 2 out of 3 patients with vestibular EH regression. On the other hand, only in two patients with cochlear EH reduction was a stable vestibular EH degree visualized, as in the others, perilymphatic enhancement of the vestibule prevented a reliable assessment of the endolymphatic space. Due to the recurrent attacks, the membranous labyrinth may be irreversibly damaged, and the linear regression of the EH may not be possible. However, the results of the present study cannot fully verify that hypothesis because of the unknown origin of the increased perilymphatic enhancement in the vestibule in nearly half of the study group. To our knowledge, no previous studies have reported a similar phenomenon visualized in an MRI. Thus, that can result from surgical irritation of the inner ear or vestibular denervation. In the present study, this phenomenon was categorized as a separate MRI observation and not considered a change in EH degree because of its unknown etiology and pathophysiology.

Interestingly, in our preliminary study evaluating four patients eight months after vestibular neurectomy, postoperative increased perilymphatic enhancement was visualized in two patients, affecting mainly the posterior arm of the lateral semicircular canal. In contrast, in the cochlea, a postoperative reduction of asymmetrical contrast enhancement was found in one patient [39]. Increased perilymphatic enhancement of the cochlea is considered an early radiological sign of MD, probably caused by the blood–perilymph barrier damage [6]. Hypothetically, changes in the blood–perilymph barrier permeability may occur due to surgical denervation of the vestibule. However, further studies on a larger group of patients are necessary to analyze the endolymphatic space of the vestibule after neurectomy. Considering increased vestibular perilymphatic enhancement as a change in the endolymphatic space volume caused by perilymphatic compression, it may be related to the EH regression. However, it would be necessary to verify the temporary character of this phenomenon and reevaluate vestibular EH. Particularly considering that postoperative regression in the cochlear EH separately and analyzing all the radiological signs was statistically significant, while reduction of the vestibular EH analyzed separately was not statistically significant. Further studies on a larger group of patients are necessary to analyze the endolymphatic space of the vestibule after neurectomy. Nevertheless, we believe that describing this radiological phenomenon may have a role in understanding hydrops pathogenesis and may be helpful in further investigations.

Although we hypothesize that regression of the EH would less likely occur in patients with longer disease duration due to the mechanical damage and loss of elasticity of the membranous labyrinth, in the present study, a longer disease duration characterized patients with partial EH withdrawal. Moreover, those groups did not differ regarding the preoperative EH degree. Several morphological changes occur in the inner ear during MD’s natural course. Recently, Pender [42] proposed a rupture risk envelope concept to explain EH development using the viscoelastoplastic properties of the endolymphatic membranes of the cochlea and the vestibule. Continuous dilatation of the membranous labyrinth may lead to irreversible loss of function of the Bast’s valve, as recently described by Buki et al. [43], who visualized in the histopathological examination that Bast’s valve was open in all patients with utricular hydrops. Thus, long disease duration may lead to the utriculosaccular valve regurgitation analogously to the heart valvular regurgitation in dilated cardiomyopathy. That process could presumably facilitate endolymph flow toward the vestibular aqueduct and lead to EH regression after reducing endolymph production caused by the dark cells’ denervation. It should be emphasized that it is only a hypothesis, and further radiological and histopathological studies are required to understand that process better.

The middle fossa approach enables hearing preservation as it does not involve the cochlear nerve section. As shown in the literature, hearing preservation is achievable in 76–98% of patients with hearing levels deteriorated no more than 10 dB compared to the preoperative level [35, 36]. However, during a long follow-up, hearing deterioration is observed in 41–48% of patients, where hearing worsened by ≥ 10 dB in 45% of cases [35, 44], which is also consistent with our study. According to one of the hypotheses, vestibular neurectomy remains a symptomatic treatment not aimed at reducing EH. Thus, endolymphatic space continues to expand after the vestibular neurectomy, consequently deteriorating hearing. However, in our study, no progression of the EH degree was observed in the postoperative MRI scans, neither in patients with hearing loss progression nor in patients with constant levels of hearing, which is not consistent with that theory.

Interestingly, in 5% (one patient, #4), hearing improvement of 20 dB was observed after the surgery, and postoperative MRI scans revealed complete cochlear EH regression from grade 2 to 0. Nevertheless, the second patient who showed complete reduction of the severe cochlear EH had postoperative hearing deterioration of 25 dB (#15), and the statistical analysis showed that patients with postoperative cochlear EH regression and the rest of the group did not differ in terms of pure-tone audiometry results. Another hypothesis is that postoperative hearing loss progression may be caused by the cochlear nerve irritation during the separation of the vestibular branch in the internal auditory canal. A longitudinal study by Li et al. [12] described changes in EH degree and PTA level in patients hospitalized twice in 1–44 months intervals. Interestingly, no differences in EH severity in the affected ear were visualized despite significant deterioration of the average PTA level. Considering all of this, we cannot assume that the progression of hearing loss results directly from the EH. Other than endolymphatic space dilatation processes might contribute to hearing loss progression in patients with MD.

## Conclusions

Vestibular neurectomy is an effective surgical method, eliminating vertigo attacks and improving quality of life in patients with clinically advanced MD. Dedicated protocol for EH visualization in the MRI examination provides new possibilities for evaluating patients with MD after conservative and surgical treatment. In the present study, regression of the EH after vestibular neurectomy was described for the first time in the literature. Although it is known to be an effective symptomatic treatment of vertigo attacks, vestibular neurectomy may also effectively halt the progression of the endolymphatic space dilatation and result in hydrops regression. Further studies are required to investigate postoperative increased perilymphatic enhancement in the vestibule, which would also be important for studying the pathogenesis of the disease.
